# The Diet Quality of Competitive Adolescent Male Rugby Union Players with Energy Balance Estimated Using Different Physical Activity Coefficients

**DOI:** 10.3390/nu8090548

**Published:** 2016-09-07

**Authors:** Tracy Burrows, Simon K. Harries, Rebecca L. Williams, Cheryl Lum, Robin Callister

**Affiliations:** 1 School of Health Sciences, Faculty of Health and Medicine, The University of Newcastle, Callaghan 2308, NSW, Australia; Tracy.Burrows@newcastle.edu.au (T.B.); Rebecca.Williams@newcastle.edu.au (R.L.W.); cheryllum1610@gmail.com (C.L.); 2 School of Biomedical Sciences and Pharmacy, Faculty of Health and Medicine, The University of Newcastle, Callaghan 2308, NSW, Australia; Simon.Harries@uon.edu.au; 3 Priority Research Centre in Physical Activity and Nutrition, University of Newcastle, Callaghan 2308, NSW, Australia

**Keywords:** nutrients, food frequency questionnaire, rugby, adolescents

## Abstract

Objectives: The aims of the current study were to comprehensively assess the dietary intakes and diet quality of a sample of Australian competitive adolescent rugby union players and compare these intakes with National and Sports Dietitians Association (SDA) Recommendations for adolescent athletes. A secondary aim investigated applying different physical activity level (PAL) coefficients to determine total energy expenditure (TEE) in order to more effectively evaluate the adequacy of energy intakes. Design: Cross-sectional. Methods: Anthropometrics and dietary intakes were assessed in 25 competitive adolescent male rugby union players (14 to 18 years old). Diet was assessed using the validated Australian Eating Survey (AES) food frequency questionnaire and diet quality was assessed through the Australian Recommended Food Score. Results: The median dietary intakes of participants met national recommendations for percent energy (% E) from carbohydrate, protein and total fat, but not carbohydrate intake when evaluated as g/day as proposed in SDA guidelines. Median intakes of fibre and micronutrients including calcium and iron also met national recommendations. Overall diet quality was classified as ‘good’ with a median diet quality score of 34 (out of a possible 73); however, there was a lack of variety within key food groups including carbohydrates and proteins. Non-core food consumption exceeded recommended levels at 38% of the daily total energy intake, with substantial contributions from takeaway foods and sweetened beverages. A PAL coefficient of 1.2–1.4 was found to best balance the energy intakes of these players in their pre-season. Conclusions: Adolescent rugby players met the percent energy recommendations for macronutrients and attained an overall ‘good’ diet quality score. However, it was identified that when compared to specific recommendations for athletes, carbohydrate intakes were below recommendations and these players in their pre-season reported high consumption of non-core foods, particularly sugar sweetened drinks and low intakes of vegetables.

## 1. Introduction

Adolescence is a life stage where dietary requirements for energy, protein, carbohydrates and other nutrients such as iron, zinc, and calcium are increased [1]. Meeting these dietary needs is important for adolescent growth, development and overall health, including protection against chronic disease [1]. This life stage is of interest from a dietary perspective as ambiguity often exists for calculating an adolescent’s requirements, as this stage covers a diverse age range and requirements can be highly variable. Adolescents who participate in regular exercise training and sports competition may have additional nutrient needs to meet their increased energy expenditure, muscle development and maintenance, as well as performance and recovery requirements [2]. In recognition of these unique needs Sports Dietitians Australia (SDA) published a Position Statement, “Sports Nutrition for the Adolescent Athlete” [2], which provides nutrition recommendations for this population. These recommendations reinforce the importance of eating for long-term health, as well as meeting specific diet and hydration needs related to exercise. SDA guidelines specifically recommend adequate intakes of calcium and iron due to an elevated risk of deficiency of these nutrients, and that these nutrient needs should be met by food rather than supplement sources [2]. The SDA statement is directed at two groups of athletes: Active adolescent athletes and competitive adolescent athletes, but not elite athletes. The competitive group are those with demonstrated sports talent who are engaged in higher volumes of training and competition. The Australian Institute of Sport, which is the leading sports training facility in Australia, also provides dietary recommendations suggesting athletes should consume a regular spread of high quality protein foods to supply the body with appropriate quantities of essential amino acids, and high quality carbohydrate foods defined as those that are nutrient dense with consideration given to glycaemic index [3].

Rugby union is an intermittent field-based team sport, involving repeated short bursts of high intensity activity interspersed with longer periods of low intensity activity [4]. Rugby union players require high levels of strength and power to perform activities such as running, sprinting, tackling, and pushing or competing for the ball. Some positions, for example rugby forwards, require a larger body mass, with higher levels of muscle mass, but these players generally have a higher percentage of body fat. Elite rugby union players are taller, heavier and have lower body fat levels than their sub-elite counterparts [4].

The nutritional demands of rugby players vary across the game’s seasons (pre-season, competitive, and off-season) [5] and likely differ depending on playing position [6]. In addition to the variations in energy needs of adolescents due to age, gender, growth and maturation, the varying levels of energy expenditure across a year make it challenging to estimate the physical activity levels (PAL) for use in estimating daily total energy expenditure (TEE) to determine the adequacy of dietary energy intake. Few studies have specifically investigated dietary intakes in adolescent rugby players. Existing studies do not comprehensively report dietary intakes; studies have examined short-term dietary intake such as single-meal consumption after training [7], reported on major or selective food groups or nutrients [8,9], and identified poor nutritional knowledge relating to sports performance [10]. Both the Australian Dietary Guidelines (ADG) and the SDA position statement recommend variety in the daily diet of adolescent athletes; however, to date no studies have investigated overall diet quality in this population group.

The aims of the current study were to comprehensively assess the dietary intakes and diet quality of a sample of Australian competitive adolescent rugby union players and compare these intakes with National and SDA recommendations for adolescent athletes. A secondary aim investigated applying different PAL coefficients to determine total energy expenditure (TEE) in order to more effectively evaluate the adequacy of energy intakes.

## 2. Participants and Methods 

### 2.1. Participants 

Competitive adolescent male rugby players (aged 14 to 18 years) were recruited from two sub-elite representative rugby union squads from the Hunter region, NSW, Australia. Sample size was based on the power to detect differences between groups for changes in box squat performance, which was the primary outcome measure of this study [11]. Data collection took place at the beginning of the pre-season period in February 2012. Ethics approval for this study was obtained from the University of Newcastle Human Research Ethics Committee and all participants provided written informed consent; parental consent was also provided. The study was registered with the Australia and New Zealand Clinical Trials registry (ACTRN12612000278831).

### 2.2. Anthropometric Assessments

These were conducted by trained research assistants between 9:00 a.m. and 10:30 a.m. during a morning assessment session at the University of Newcastle. Participants were advised with consistent information to eat a light breakfast and to perform no exercise training on the day before assessments. Height was recorded using a calibrated stadiometer (Harpenden portable stadiometer with high speed Veeder-Root counter, Holtain Ltd., Pembrokeshire, UK) and bodyweight determined using calibrated scales (CH-150kp, A&D Mercury Pty Ltd., Seven Hills, NSW, Australia). Repeat assessments were performed to ensure accuracy of measures. If there was a difference of 0.3 cm or 0.1 kg between the two measurements, a third measure was taken. Body Mass Index (BMI) was calculated using standardised equations and BMI *z* score calculated using Lambda, Mu, Sigma (LMS) methods [12]. Body composition was determined via bio-impedance analysis using the INBODY720 Body Comp analyser (InBody720, Biospace Co., Ltd., Seoul, Korea) with body fat (kg and %), fat free mass (FFM) (kg) and skeletal muscle mass (SMM) (kg) determined. The InBody720 has been shown to display a high level of agreement with dual energy X-ray absorption spectroscopy (DEXA), the gold standard for body composition analysis, in the measurement of body fat mass (ICC males = 0.93, *p* < 0.001 [13]), and compared to computed tomography when measuring visceral fat area (*r* = 0.76) [14].

### 2.3. Dietary Intakes 

This was assessed using the Australian Eating Survey (AES), a 120-item semi-quantitative food frequency questionnaire (FFQ) validated for use in Australian children for energy and nutrient intakes, as well as fat profiles and fruit and vegetable intakes through a range of objective biomarkers [15,16,17]. This dietary assessment method was chosen over alternate methods as it has a longer reporting period and is more likely to capture usual/habitual dietary intakes. An individual response for each food is required, with consumption frequency options ranging from ‘Never’ to ‘4 or more times per day’ and for some beverages up to ‘7 or more glasses per day’, but varied depending on the item. The AES groups items according to their food group as follows: Core foods which include breads and cereals, fruit, vegetables (including potatoes), dairy, meat and meat alternatives (legumes, nuts, eggs, tofu), and non-core foods which include those foods characterised as high in sugar, salt or fat, such as sweetened drinks, packaged snacks, confectionary and takeaway foods. Fifteen supplementary questions assessed dietary behaviours including use of vitamin supplements (dosage and length of use) and frequency of take-out food.

### 2.4. Diet Quality

The quality of diet was assessed through the Australian Recommended Food Score (ARFS), which is a validated food-based diet quality index modelled on the Recommended Food Score by Kant and Thompson [18,19] and the Australian Child and Adolescent Recommended Food Score (ACARFS) [20,21]. The ARFS focuses on dietary quality and variety within food groups recommended in the Australian Dietary Guidelines (ADG) [1]. It is calculated by using a subset of 70 AES FFQ questions. The ARFS has sub-scales of food groups including fruits, vegetables, grains, protein sources, vegetarian protein sources, dairy and condiments. Most foods are awarded one point for a consumption frequency of ≥once per week, but varies based on national dietary guidelines [1,22] with bonus points for grained varieties of breads and cereals and low fat dairy. The ARFS score was calculated by summing the points for each item, the total score ranges from zero to 73.

Nutrient intakes were computed using the Australian AusNut 1999 database (All Foods) Revision 17 primarily, and AusFoods (Brands) Revision 5 (Australian Government Publishing Service, Canberra, Australia). The estimated mean individual daily intakes for macro- and micro-nutrients were calculated using FoodWorks (version 3.02.581, Xyris Software, Highgate Hill, Queensland, Australia). The computed data was then compared to standardized national data [23]. To standardise the assessment of fibre intakes for participants with differing energy intakes, grams of fibre/1000 kJ were calculated. Servings of fruits and vegetables were calculated by summing the weight or energy of food items in the AES coded as fruits or vegetables and dividing by the serve size dictated in the Australian Guide to Healthy Eating (AGHE) (fruits, 150 g and vegetables, 75 g, grains, meat/alternatives and dairy 500–600 kJ/serve). All other foods were quantified using multiples of standard child portions from the 1995 Australian National Nutrition Survey of children and adolescents, which are suitable for use in this population group. 

### 2.5. Physical Activity Level (PAL) Calculations

In order to determine whether energy intakes met energy needs, equations were used to predict exercise and total energy expenditure (TEE) and from this calculate energy availability. The Schofield equation using both height and weight values is the preferred equation for estimating resting energy expenditure (REE) in children and adolescents [24]. To better estimate TEE, a PAL coefficient can be applied to account for exercise related increases in EE. PAL recommendations are available for adult athletes however there is a lack of consensus of PAL levels to use in adolescent athletes [25,26,27]. For the purpose of this study, a range of coefficient values were applied to calculated REE to reflect a range of activity levels. These included: No PAL, and PAL 1.2, 1.4, 1.6 and 1.8.

### 2.6. Data Analysis

Participant demographics and data from the food frequency questionnaires were analyzed using SPSS. Data were not normally distributed so non-parametric tests were used and data are presented as median and interquartile range. Wilcoxon rank tests were used to compare differences between estimated energy needs and reported energy intake. Bland Altman plots were produced according to standardised methods [28] to assess levels of agreement between reported and estimated energy requirements. Statistical significance was set at the 5% level (*p* < 0.05).

## 3. Results

### 3.1. Participant Characteristics

Participant characteristics are reported in Table 1. The median (IQR) age of players was 16 (2) years with a BMI of 23.6 (5.5) kg/m^2^ Body composition analysis showed body fat was 11.7 (6.2) kg, fat free mass was 66.1 (13.7) kg and skeletal muscle mass was 37.9 (9.9) kg. Participants reported that, on average, they were currently doing one hour or less per day of exercise.

### 3.2. Energy Needs and Energy Intakes

The median (IQR) BMR of the rugby players was 8379 (1254) kJ and total estimated energy requirements ranged from 10,055 (1505) kJ to 15,083 (2258) kJ when using a PAL of 1.2 up to 1.8, respectively. Reported dietary intakes as per the AES FFQ are summarised in Table 2 and Table 3. The median (IQR) reported energy intake was 10,372 (4974) kJ.

Applying a PAL coefficient of 1.2 or 1.4 produced the closest TEE for energy balance between the reported intakes and calculated expenditures. Using a PAL of 1.2 or 1.4, the mean differences between TEE and reported energy intakes were −614 kJ (*p* = 0.480) and +1129 kJ (*p* = 0.209) respectively. Applying no PAL or the larger coefficients of 1.6 and 1.8, reflecting more extensive exercise, provided larger discrepancies with differences of 2872 kJ (*p* = 0.004) and 4516 kJ (*p* < 0.000) for PAL 1.6 and 1.8, respectively. 

### 3.3. Macronutrients: Fats, Protein and Carbohydrates

The median intake of all participants met the recommended percentage of daily total energy intake for Australians (% TE) from macronutrients for energy derived from carbohydrate (45%–65% TE), total fat (20%–35% TE) and protein intakes (15%–25% TE) (Table 2). Reported intakes of saturated fats (15% E) exceeded the national recommended intake of <10% TE, with the median intake of 15% TE with 4% of total energy intake from poly-unsaturated fat and 12% from monounsaturated. 

Protein intakes in this study were found to be similar when compared to the Australian Health Survey (AHS) data of 14- to 18-year-old males in the general population and were within 4% of each other [24]. When intakes for this study were compared directly to SDA recommendations, protein requirements in g/kg were also met with the median intake of 1.5 g/kg/day.

Carbohydrate intakes in this study were within 7% of those averages found for adolescent males in the AHS [23]. When expressed as g/kg, as recommended by the SDA for carbohydrates, the median intake of this population was below that suggested with a median intake of 3.6 g/kg compared to the recommended 5–7 g/kg/day for those following a moderate exercise program [2,3].

### 3.4. Micronutrients 

Intakes of iron and calcium met both National Dietary Recommendations (Table 2) and SDA recommendations. Intakes of calcium and iron were higher in rugby players in this study than in adolescent males of the same age in the general Australian population (AHS data) [23] with a calcium intake approximately 200 mg higher (approximately 0.8 serve/day) and iron intake 2 mg higher. The fibre intake met recommendations assessed both as grams per day (RDI 28 g/day) and when adjusted for energy intake as g/1000 kJ. The fibre intake of these males was on average 5 g/day higher than that of the general population who had an intake of 23 g/day [29].

### 3.5. Fluid Intake

Only 23% of adolescents reported consuming >seven glasses of water/day. Consumption of sugar-sweetened beverages was deemed high with soft drinks (not diet), fruit juice-based drinks and cordial (make up) consumed at intakes >two glasses/day by 26%, 44% and 23% of participants, respectively. Fruit juice-based drinks were the most commonly consumed sweetened drink in these players. Vitamin supplements were reported as being consumed by only 8% (*n* = 2) of the participants, at a dose of three to five vitamin tablets per week. 

### 3.6. Food Groups and Diet Quality

Table 3 presents the dietary intakes from major food groups and food subgroups as well as the total diet quality score for both rugby players in this study and males of the same age in the general population. The median % TE derived from core foods for males in this study (breads and cereals, vegetables, fruits, meat and meat alternatives, milk and dairy) was 62% (IQR 19) and for males in the general population it was 59%. The reported number of servings of fruit (which excludes juice) was 4.7 servings/day for males in this study, which exceeded the AGHE recommendation of two servings/day. Only 46% of male children and adolescents in the general population consume the recommended two servings/day, consuming a mean of 1.5 servings and median 1.1 servings [30]. Intakes of vegetables in these adolescent rugby players were well below recommendations at 1.1 servings/day compared to the recommendation of five servings/day. Males of the same age in the general population also had suboptimal intakes of vegetables consuming a mean of 2.2 (median 2.1) servings/day. Intakes of grains were 3.7 servings/day in this study and a mean of 5.7 (5.5 median) servings/day in males in the general population, with both well short of the recommended seven servings/day. Meat/alternatives in the rugby players at 3.4 servings/day was higher than in the general population (1.7 servings/day). Neither the males in this study nor in the general population met the 3.5 servings/day recommendation for dairy intake, consuming 2.1 and 1.6 servings/day, respectively.

The median % TE derived from non-core foods in this study was 38% (IQR 19) and that of males in the general population was 40.7%, both of which exceed the recommendation of 5%–10% of the total energy intake. The main sources of non-core food energy in these rugby players were 4.4% TE from sweetened drinks (soft drinks/cordials/sports drinks/juice), 5.4% TE from packaged snacks (muesli/snack bars), 3.8% TE from confectionary (chocolates/candy), and 8.1% TE from takeout meals, which includes hamburgers, fries, pies and sausage rolls.

### 3.7. Diet Quality Scores

The median ARFS score was 34 out of a possible 73 points, which is classified as ‘good’ (range 32 or above) diet quality [28]. A breakdown into the ARFS subgroups identified a lack of variety within subgroups, with median scores for vegetables at 12 out of a possible score of 21, fruit at six out of 12, dairy at 4.5 out of 11, meat at three out of seven and meat/alternatives at two out of six.

## 4. Discussion

This study investigated the dietary intakes and diet quality of competitive adolescent male rugby players using a validated FFQ. These players had adequate macronutrient profiles for carbohydrate, total fat and protein intakes but exceeded national recommendations for saturated fat. Micronutrient intakes, including calcium and iron, were also adequate. Excess energy was derived from non-core foods, particularly fruit juices and other sweetened drinks, and there was inadequate vegetable consumption when compared to national recommendations. Overall diet quality was classified as ‘good’, although the results indicate a substantial scope to improve diet variety, particularly within the fruit, vegetable and dairy food groups. 

The energy requirements of the adolescent athletes were met by their dietary intakes when a PAL coefficient of 1.2 or 1.4 was used. It is important to recognise that these players were assessed immediately prior to the commencement of pre-season training. The period over which they reported their diet was the off-season. Players reported less than one hour of physical activity a day during this period. This suggests that they may need to increase their energy intake once training and then playing commence, and that the PAL required to calculate energy needs would likely approach 1.6 for this period of the year. 

Macronutrients: Protein intakes were found to be meeting and/or exceeding SDA recommendations in competitive adolescent rugby players, for both percentage of total energy intake and g/kg. For carbohydrate intakes, this population group was found to have adequate intakes compared to national dietary recommendations for percentage of total energy intake. However, when compared to SDA recommendations for carbohydrate intake expressed as g/kg, their intakes were found to be inadequate for those undertaking moderate exercise training. Given these players were just commencing their pre-season, their physical activity levels were likely lower than they would become during pre-season training and the competition season where their estimated requirements for carbohydrate could increase to as much 6–10 g/kg [3]. Total fat intakes were found to be within recommended ranges for this age group; however, saturated fats exceeded recommendations. This is likely to have been largely derived from non-core foods which also exceeded recommendations.

Micronutrients: Recommended intakes were met for calcium and iron, which were a focus in this study as they are specifically mentioned in the SDA position statement as important to adolescent health. Although the players did not meet the recommended servings of dairy foods per day, which are recognised as excellent sources of calcium, these players still met their calcium requirements, indicating they are likely deriving calcium from alternate food sources including nuts and beans. These athletes did not rely on vitamin supplements to meet these nutrient needs, with few participants reporting regular intake. Dietary intakes of fibre were met, which provides some indication of the quality of the carbohydrate intake of these rugby players. In this study, fibre intake was adjusted for energy intake and reported per 1000 kJ so it is not simply reflective of higher energy intake. Fibre intake is likely to be derived from carbohydrate-based sources from whole grains including breakfast cereals often containing moderate amounts of fibre which are more nutrient dense than refined flour sources. Servings of fruit were adequate but not vegetable intakes. Increased intakes of vegetables are associated increased intakes of nutrients. It is recommended that intakes of vegetables be increased in these adolescent athletes to ensure intakes of other vitamins and minerals such as magnesium for muscle function [31] and antioxidants for inflammation [32].

The consumption of non-core foods was higher than desirable, with energy from takeout and sugar-sweetened beverages being the major contributors. Participants consumed low quantities of water compared with fluid intakes from other sources. Sweetened drinks including soft drinks and cordial but particularly fruit juice-based drinks were commonly consumed; sports drinks were not directly assessed in this study. This finding may be reflective of the broader food environment where current food trends such as smoothies and juice bars increase the availability and perception that these drinks are healthy [33]. It is acknowledged that increased consumption of high-energy-density items such as sweetened drinks may help in achieving the energy needs of adolescent athletes; however, this may also have implications for excess energy intake and oral health [34]. Energy-dense items may also be considered convenient for adolescents and might characterise adolescent diets, which are increasingly influenced by peers and the media, as they start to assert independence and control of their food intakes and preferences [35,36]. This is consistent with other research where adolescents consume intakes of energy-dense nutrient-poor foods well above the national recommendation of 5% of energy. In this study, non-core foods were found to contribute approximately 38% of daily energy, which is consistent with national dietary surveys where reported intakes constitute up to 41% of daily energy [37]. Elite rugby athletes are required to have speed, agility, strength and power; this high consumption of non-core foods may compromise the players’ body composition, fitness, and performance [38].

Both the Australian Dietary Guidelines (ADG) and the SDA position statement recommend variety in daily diet. The SDA recommendations particularly emphasise the quality of carbohydrate and protein foods for adolescent athletes [2]. This is consistent with the broader ADG, which recommend variety in daily diet in addition to variety within food groups, as this is more likely to produce a diet with a more comprehensive and complete range of nutrients. Quality carbohydrate foods generally include those that are not highly processed and are made from whole grains with adequate quantities of fibre. The quality of foods in this study was assessed through the ARFS, which values both food quality and variety among food groups and within groups; for example, the meat and alternatives group includes a range of foods such as meat, eggs, and fish, all of which differ in their nutrient profiles. Although the overall ARFS score for the players’ diets was considered good, the scores of important food subgroups such as fruit, vegetables, dairy and protein foods were less than 50% of the available points, which reflects a lack of variety within these food groups [28]. Improvements in consumption of these foods could be targeted in future interventions. 

Studies suggest adolescents consume supplements for health benefits, energy and enhancement of sports performance [39,40,41]. Common supplements used by athletes include sports drinks, protein powders and creatine; however, there was very little use of supplements in this study. Less than 10% of players reported consuming vitamin supplements, which was less than expected [39]. Recent studies which used a four-day food diary to assess dietary intake reported that 74% of 14- to 19-year-old rugby players consumed dietary supplements [9]. Details on the assessment of supplements used by participants in this study were limited to consumption patterns and frequency of intake; the types of supplements (i.e., protein or antioxidants) and reasons for use were not assessed. These should be investigated more thoroughly in future studies.

Nutrition education has been identified as an area of need for adolescent athletes previously in the literature [10], especially for adolescent rugby players. While previous studies did not describe areas for nutrition education, the results from this study provide a starting point for areas which require improvement to improve overall health and performance. These include education on food groups, particularly non-core foods (sweetened drinks, takeaways), as a strategy to decrease overall saturated fat and optimise fat profiles, and the importance of overall diet quality and diet variety within food groups to ensure adequacy of diet.

The AES FFQ used in this study has been validated against a number of objective dietary standards including double-labelled water, plasma carotenoids and red blood cell membrane fatty acids; however, it is acknowledged that FFQs, like most dietary assessment tools, are prone to bias, being a self-report measure [42]. The FFQ used assesses the usual dietary intake with a reporting period of the previous six months; there is no assessment of the timing of intake. Studies in athletes [40] demonstrate that timing of dietary intake is an important factor to maximise performance and should be assessed in future studies through use of a diet history, a food and training diary or direct observations. Timing of meals and snacks may be particularly important for nutrients such as protein and should be investigated in future studies, as an amount of approximately 20 g of protein throughout or immediately following strength training enhances acute protein synthetic responses to the training stimulus [43]. Body composition in this study was measured using an INBODY720 Body Composition Analyzer and not the recognised gold standard DEXA method for assessing body composition. Other limitations to the study include the small sample size, estimation of energy expenditure through a standardised equation rather than measurement of resting energy expenditure, and that the population studied was from one country and two squads from the same region; while all participants were advised to have light breakfast before assessments, there may have been some variation in intake prior to assessments. Future studies investigating dietary habits in competitive adolescents should consider a multi-centre trial and assessing diet during different stages of the training and competitive seasons. 

## 5. Conclusions

This study examined the nutrient profiles and diet quality of competitive adolescent rugby players. The key findings were that these players met the percent energy recommendations for macronutrients and attained an overall ‘good’ diet quality score. However, it was identified that when compared to specific recommendations for athletes, carbohydrate intakes were below recommendations and these players in their pre-season reported high consumption of non-core foods, particularly sugar-sweetened drinks, and low intakes of vegetables, highlighting particular areas for dietary education. During the off-season, a PAL of 1.2 or 1.4 appears appropriate for determining energy balance; however, further research should investigate the use of PALs at different training loads.

## Figures and Tables

**Table 1:** Participant characteristics of adolescent rugby players.

Characteristic	Median (IQR)
Age (year)	16 (2)
Weight (kg)	76.5 (10.0)
Height (cm)	179.6 (6.5)
Body fat (kg)	11.7 (6.2)
Body fat (%)	14.0 (6.6)
Fat free mass (kg)	66.1 (13.7)
Skeletal Muscle Mass (kg)	37.9 (9.9)
Basal Metabolic Rate (/day)	
kJ	8379 (1254)
kCal	1995 (299)
Estimated Energy Requirement (kJ)	
PAL 1.2	10,055 (1505)
PAL 1.4	11,731 (1756)
PAL 1.6	13,407 (2007)
PAL 1.8	15,083 (2258)
Estimated Exercise Expenditure (kJ)	
PAL 1.2	1676 (251)
PAL 1.4	3352 (502)
PAL 1.6	5028 (753)
PAL 1.8	6704 (1003)

**Table 2:** Energy and nutrient intakes of adolescent male rugby players and comparison with National Dietary Recommendations and SDA recommendations.

Nutrient	Median (IQR)	% Total Daily Energy	Health Recommendations [1]	SDA Recommendations [2]
Energy (kJ)	10,372 (4974)	X	X	
Protein (total g)	108.1 (75.9)	19 (5)	15%–25% of energy	
g/kg/day	1.53 (0.86)	X	0.8 g/kg	1.3–1.8 g/kg
Fat (g)	88.5 (54.9)	34 (8)	20%–35% energy	
Saturated fat (g)	39.8 (21.5)	15 (4)	8%–10% of energy	
Polyunsaturated fats (g)	9.4 (6.6)	4(1)		
Monounsaturated fats (g)	29.9 (16.4)	12 (3)		
Carbohydrates (g)	317 (153)	48 (12)	45%–65% of energy	
g/kg/day	3.59 (2.40)	X	X	5–7 g/kg
Sugars (g)	149.4 (117.4)	X	X	
Fibre (g)	33.4 (15.1)	X	28 g/day	
Fibre/1000 kJ	2.6 (0.6)	X	X	
Calcium (mg)	1124 (713)	X	1050 mg/day	
Iron (mg)	15.9 (7.9)	X	8 mg/day	

EAR—Estimated average requirements, ARFS—Australian Recommended Food Score: A measure of diet quality, X no specific recommendations/NA.

**Table 3:** Food group intakes of adolescent male rugby players.

Food Sub Groups	Population				Health Recommendations (Servings/Day) [1]
	14–18 Years Old Rugby Players		14–18 Years Old Males—AHS		
	Median (IQR) (Servings/Day)	% Total Daily Energy	Mean (Servings/Day)	% Total Daily Energy	
**Fruit **	4.7 (5.0)	X	1.5	X	2
**Vegetables **	1.1 (0.5)	X	2.2	X	5 ½
**Grains **	3.7 (2.8)	X	5.7	X	7
**Meat/alternatives**	3.4 (2.5)	X	1.7	X	2 ½
**Dairy **	2.1 (2.1)	X	1.6	X	3 ½
**Discretionary foods**	7.5 (3.7)	38.4	X	40.7	0–5 *
**Energy (kJ): sweetened drinks **	508 (616)	4.4 (8.2)			X
**Energy (kJ): packaged snacks **	549 (780)	5.4 (7.8)			X
**Energy (kJ): confectionary **	376 (516)	3.8 (4.8)			X
**Energy (kJ): takeaway **	913 (521)	8.1 (4.8)			X
**Diet Quality**	**Score**				
	**14–18 Years Old Rugby Players**		**Adults (18+ Years)**		
**Total ARFS (out of 73)**	34 (13)	X	36 (10.5) [21]		X

AHS—Australian Health survey; % TE—percentage of total energy; * Approximate number of additional servings from the five food groups or unsaturated spreads and oils or discretionary choices.
